# Hookworm Infection and Environmental Factors in Mbeya Region, Tanzania: A Cross-Sectional, Population-Based Study

**DOI:** 10.1371/journal.pntd.0002408

**Published:** 2013-09-05

**Authors:** Helene Riess, Petra Clowes, Inge Kroidl, Dickens O. Kowuor, Anthony Nsojo, Chacha Mangu, Steffen A. Schüle, Ulrich Mansmann, Christof Geldmacher, Seif Mhina, Leonard Maboko, Michael Hoelscher, Elmar Saathoff

**Affiliations:** 1 Division of Infectious Diseases and Tropical Medicine, Medical Center of the University of Munich, Munich, Germany; 2 Institute for Medical Bioinformatics, Biometry, and Epidemiology, Ludwig-Maximilians-University, Munich, Germany; 3 NIMR-Mbeya Medical Research Center, Mbeya, Tanzania; 4 German Centre for Infection Research (DZIF), Partner Site Munich, Munich, Germany; 5 Mbeya Regional Medical Office, Mbeya, Tanzania; University of Melbourne, Australia

## Abstract

**Background:**

Hookworm disease is one of the most common infections and cause of a high disease burden in the tropics and subtropics. Remotely sensed ecological data and model-based geostatistics have been used recently to identify areas in need for hookworm control.

**Methodology:**

Cross-sectional interview data and stool samples from 6,375 participants from nine different sites in Mbeya region, south-western Tanzania, were collected as part of a cohort study. Hookworm infection was assessed by microscopy of duplicate Kato-Katz thick smears from one stool sample from each participant. A geographic information system was used to obtain remotely sensed environmental data such as land surface temperature (LST), vegetation cover, rainfall, and elevation, and combine them with hookworm infection data and with socio-demographic and behavioral data. Uni- and multivariable logistic regression was performed on sites separately and on the pooled dataset.

**Principal Findings:**

Univariable analyses yielded significant associations for all ecological variables. Five ecological variables stayed significant in the final multivariable model: population density (odds ratio (OR) = 0.68; 95% confidence interval (CI) = 0.63–0.73), mean annual vegetation density (OR = 0.11; 95% CI = 0.06–0.18), mean annual LST during the day (OR = 0.81; 95% CI = 0.75–0.88), mean annual LST during the night (OR = 1.54; 95% CI = 1.44–1.64), and latrine coverage in household surroundings (OR = 1.02; 95% CI = 1.01–1.04). Interaction terms revealed substantial differences in associations of hookworm infection with population density, mean annual enhanced vegetation index, and latrine coverage between the two sites with the highest prevalence of infection.

**Conclusion/Significance:**

This study supports previous findings that remotely sensed data such as vegetation indices, LST, and elevation are strongly associated with hookworm prevalence. However, the results indicate that the influence of environmental conditions can differ substantially within a relatively small geographic area. The use of large-scale associations as a predictive tool on smaller scales is therefore problematic and should be handled with care.

## Author Summary

Hookworm disease, caused by the nematodes *Ancylostoma duodenale* and *Necator americanus*, is an important cause of maternal and child morbidity in the developing countries of the tropics and subtropics. In children, hookworm disease has been shown to potentially result in growth retardation as well as intellectual and cognitive impairments. In a cross-sectional survey in Mbeya region, Tanzania, we assessed the effects of possible risk factors for hookworm infection with a focus on remotely sensed ecological factors such as elevation, vegetation density, land surface temperature, and rainfall. We found that several ecological variables were significantly associated with hookworm infection. However, differing effects for these factors were estimated when performing the analyses separately for the two sites with the highest hookworm prevalence. Our study shows that effects are scale-dependent and that prediction at smaller scales using large-scale data and *vice versa* should be handled with caution, because regional variation can substantially influence the presence of hookworm infection.

## Introduction

Hookworm disease caused by *Ancylostoma duodenale* and *Necator americanus* is among the most common infections in sub-Saharan Africa (SSA) and affects up to 198 million people in this region [1]–[3]. It causes iron deficiency anemia and protein malnutrition, and has been shown to potentially cause growth retardation as well as intellectual and cognitive impairments in children [2]–[4]. Although hookworm disease causes only limited mortality, it ranks 49^th^ in terms of years lost due to disability globally and between 30 and 49 in SSA countries [5]. The educational, economic, and public-health importance of hookworm infection necessitates comprehensive control strategies. To assure the effectiveness of control programs, financial as well as human resources have to be targeted to areas of greatest need. This warrants reliable estimates of hookworm distribution and of population numbers requiring intervention [6]. As hookworms do not replicate inside the human body and larvae become infective only under favorable conditions once excreted, environmental factors are crucial to hookworm development and therefore to possible transmission to humans. In recent years, the use of remotely sensed data has helped to enhance the understanding of the epidemiology and spatial distribution of hookworm infection [7], [8]. Model-based geostatistics have been used to map helminth infection prevalence and to predict prevalence at unsampled locations at national, provincial, and regional levels [6], [9]–[11], however, under the assumption that the estimated associations are the same at all levels and not modified by regional characteristics.

This study aimed to investigate the relationship between hookworm infection and remotely sensed ecological factors, such as elevation, vegetation density, land surface temperature (LST), and rainfall, at an individual level in a cross-sectional survey of the “Evaluating and Monitoring the Impact of New Interventions” (EMINI - http://www.mmrp.org/projects/cohort-studies/emini.html) cohort in Mbeya region in south-western Tanzania. Furthermore, we analyzed the influence of potential confounders, such as age, sex and socio-economic status (SES), on these associations. The main focus was on the investigation of site-specific effects and their comparison to effects in the pooled data set to ascertain if associations between ecological factors and hookworm infection found on a larger scale can equally be applied at smaller scales. Additional articles pointing to *Ascaris lumbricoides*, *Trichuris trichiura*, *Schistosoma mansoni*, and *Schistosoma haematobium* infection are in preparation.

## Methods

### Ethics Statement

The study was approved by the ethics committee of the Tanzanian National Institute for Medical Research and conducted according to the principles expressed in the Declaration of Helsinki. All participants provided written informed consent before enrolment into the study; parents consented for their minor children.

### Study Area and Data Collection

Mbeya region is situated in south-western Tanzania. The region is predominantly rural and most income-generating activities are related to agriculture. Data for this study were collected from June 2008 to June 2009 as part of the third annual survey of the EMINI cohort study. In preparation for EMINI, a complete census was undertaken in nine distinct sites of Mbeya region. Over 42,000 households were identified and their locations were georeferenced using hand-held global positioning system (GPS) receivers (SporTrak handheld GPS, Magellan Navigation Inc., Santa Clara, CA, United States of America). A geographically stratified random sample of approximately 10% of these households was selected to participate in the cohort study. During the first two EMINI surveys only blood (for HIV and *Plasmodium falciparum* malaria testing), urine (for *S. haematobium* diagnosis), and sputum samples (from participants with persistent cough for tuberculosis diagnosis) were collected. Interventions during this time included HIV and tuberculosis counseling and referral, treatment of malaria (with artemether/lumefantrine) and *S. haematobium* infections (with praziquantel).

Stool collection only started at the third annual survey, and only included inhabitants of a 50% random sample of the EMINI households. Before this survey, intestinal nematodes were neither diagnosed nor treated as part of this study, and to our knowledge no other treatment programs had been conducted in the region. Stool samples were collected in pre-labeled screw-top containers, refrigerated at 4°C directly after collection using mobile refrigerators (WAECO CoolFreeze CF-50, WAECO, Emsdetten, Germany) and kept cool until examined in the laboratory within two days of collection. The hookworm infection status of participants was established by Kato-Katz examination of two sub-samples (41.7 mg each) from a single stool specimen which was thoroughly mixed before slide preparation. Kato-Katz slides were examined for hookworm eggs by experienced staff within one hour and for other helminth eggs within two days after slide preparation. Hookworm infection was defined as the presence of at least one hookworm egg in any of the two slides. Helminth-infected participants were offered treatment with albendazole (for hookworm and other intestinal nematode infections) and/or praziquantel (for schistosome infections), according to their respective diagnoses.

Interviews were conducted to collect socio-demographic information. Age, sex, latrine type, and previous worm treatment were included as potential confounders to be adjusted for during analyses. In order to adjust for possible socio-economic confounding, we constructed an SES score using polychoric principal component analysis (PCA) [12], [13] to characterize the socio-economic situation of each household. This score combines information on the availability of certain items in the household (radio, TV, mobile telephone, refrigerator, hand cart, bicycle, motor cycle, car, savings account); sources of energy and drinking water; quality of materials used to build the main house; and number of persons per room in the household.

### Ecological Data

Information on elevation was retrieved using the NASA Shuttle Radar Topography Mission (SRTM) global digital elevation model (DEM) version 2.1 with a nominal resolution of 90 m [14]. Rainfall and ambient temperature interpolated surfaces with 1 km spatial resolution [15] were downloaded from the WorldClim – Global Climate Data website (http://www.worldclim.org/).

LST during the day (LSTday) and during the night (LST-night), and vegetation density (EVI = enhanced vegetation index) were retrieved from data collected during NASA's Moderate-Resolution Imaging Spectroradiometer (MODIS) mission and were acquired from the Land Processes Distributed Active Archive Center (LP DAAC), located at the U.S. Geological Survey (USGS) Earth Resources Observation and Science (EROS) Center [16]. LST data (version MOD11A2) have 8 days temporal and ∼1 km spatial resolution. Vegetation data (version MOD13Q1) have 16 days temporal and 250 m spatial resolution [17].

Both, LST and vegetation data were processed in the following way to produce long-term averages: data surfaces for every 8-day period (LST) and every 16-day period (EVI) for the years 2003 to 2008 were imported into Idrisi GIS software v.32 (Clark Labs, Worcester, MA, United States of America). In Idrisi, long-term averages of day- and night-LST and EVI were calculated utilizing only those pixels that were “good data” according to the quality assessment layers that are distributed together with the actual data. Then LST was converted to °C and EVI was converted back to its native range between −1 and +1. Population and household densities, ambient temperature, rainfall, LST, EVI, and elevation variables were averaged for a buffer area within a 1000 m radius around each household in order to characterize the ecological situation around the household.

### Statistical Analyses

Stata statistics software (version 11, StataCorp, College Station, TX, United States of America) was used for all statistical analyses. Some of the variables were transformed in order to yield interpretable results. Reported odds ratios (OR) for continuous variables correspond to an increase of 10 years for age, 100 m for elevation, 10 mm for mean annual rainfall, 1,000 people/km^2^ for population density, and 0.1 units for EVI.

Univariable logistic regression was performed with each variable, adjusting for within-household clustering using Huber/White/Sandwich variance estimates [18]–[20]. Variables that either had a Wald's p-value<0.2 or were considered to be causally linked to hookworm infection were included in the following selection process.

This study mainly focused on ecological data which by their nature are prone to be correlated. To avoid problems in effect estimation such as variance inflation, all variables of interest were tested for multicollinearity by calculating the variance inflation factor (VIF) [18]. A VIF above 10 was considered as an indicator for serious multicollinearity [19], [20] and the respective variables were removed from further analyses.

Subsequently, two separate logistic models were developed: the first contained solely variables collected on an individual level, i.e., age, sex, and previous worm treatment variables; the second model grouped together variables that were collected at the household level. These included environmental variables as well as the SES score, latrine coverage, and latrine type. Model selection was based on a 5% significance level, i.e., removal of variables that had p>0.05, and on the contribution to the goodness of model fit according to the Bayesian information criterion (BIC). When the removal of a variable whose effect estimate did not reach statistical significance resulted in a major increase of the BIC, the variable was not excluded from the model. Remaining variables from the separate models were merged into a final model where variables with p>0.05 were removed by hand. The resulting model was then run on a reduced dataset restricted to the two sites with the highest hookworm prevalence. To detect differences in effects on a site level, a moderated multiple regression was performed by introducing a site dummy variable as the moderator and interactions of this moderator with each environmental variables. Furthermore, the final model was applied to each of these two sites separately and compared to the results of the moderated model.

## Results

### Descriptive Statistics

**Figure 1:**
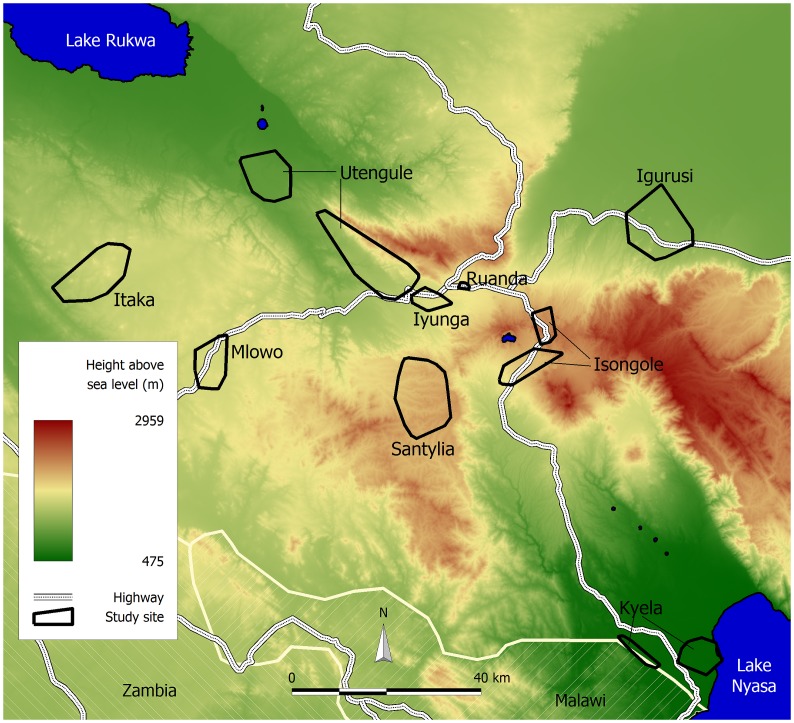
Location and altitude of the nine EMINI study sites (Mbeya region, Tanzania, 2008/2009). Elevation of the participating households ranges from 480 to 2,300 m above sea-level, resulting in large ranges also for the other environmental parameters that were examined.

**Figure 2:**
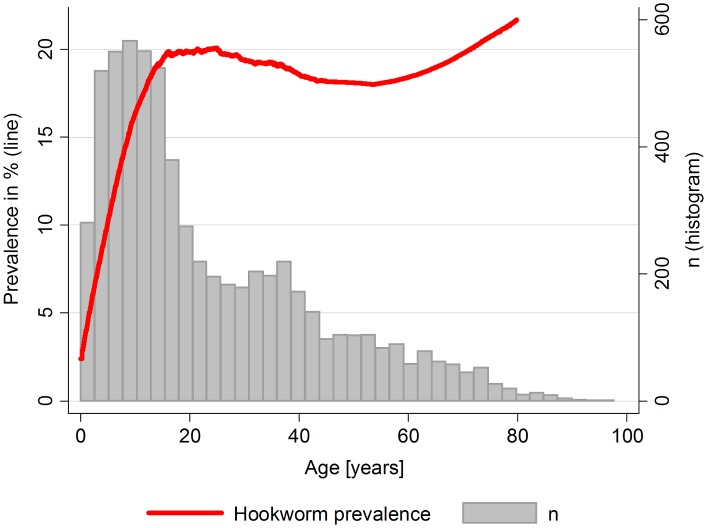
Lowess smoothed plot of hookworm prevalence over age in EMINI participants (Mbeya region, Tanzania 2008/2009). Prevalence rises sharply from birth to adolescence and reaches a plateau in early adulthood after which it stays relatively stable.

Of the 6,375 subjects (from 1,617 households) participating in this study, 17% (1,080 participants) were tested positive for hookworm infection. Most infected participants had low intensity infections (1,061), whereas medium (14) and high intensity infections (5) were rare [21]. The diverse environmental conditions in the study area are indicated by large ranges for elevation (Figure 1) and other environmental variables (Table 1). The study population included slightly more female than male participants. The median age of 16.6 years indicates that the majority of study subjects were children and adolescents. The prevalence of hookworm infection rose sharply from birth to adolescence and reached a plateau in early adulthood, after which it stayed relatively constant (Figure 2). Most households had simple or improved ventilated pit latrines, whereas water flush toilets were uncommon.

**Table 1:** Characteristics of the study participants and environmental conditions at their places of residence.

Variable	N	Median or proportion (%)*	IQR	Min	Max
Hookworm infection binary	6,375	16.9%			
Hookworm infection intensity					
Median EPG (infected only)	1,080	132	228	12	6,187
Grouped†:					
No infection (0 EPG)	5,295	83.1%			
Low intensity (1–1,999 EPG)	1,061	16.6%			
Moderate intensity (2,000–3,999 EPG)	14	0.2%			
Heavy intensity (≥4,000 EPG)	5	0.1%			
**Ecological variables**					
Elevation [m]	6,375	1,574	510	479	2,313
Mean annual EVI	6,375	0.287	0.071	0.151	0.472
Mean annual LST-day [°C]	6,375	33.2	3.4	22.5	38.6
Mean annual LST-night [°C]	6,375	14.0	4.2	9.2	21.4
Mean annual ambient temperature [°C]	6,375	19.5	3.7	14.7	25.0
Mean annual rainfall [mm]	6,375	1,254	444	1,013	2,342
Slope [°]	6,375	2.34	2.76	0.35	13.64
Population density [persons/km^2^]	6,375	415	1,745	10	13,133
**Adjustment variables**					
Age [years]	6,375	16.6	26.5	0	97.7
Sex [0 = female/1 = male]	6,326	47.0%			
SES score	6,372	−0.092	1.202	−1.892	3.997
Anthelmintic treatment in past year [0 = n/1 = y]	5,829	7.1%			
Latrine coverage in surroundings [%]	6,375	99.3	5.7	36.15	100
Latrine type in household	6,372				
None	160	2.5%			
Pit latrine simple	5,860	92.0%			
Ventilated improved pit latrine	230	3.6%			
Water flush toilet	122	1.9%			

EPG = eggs per gram of feces; EVI = enhanced vegetation index; IQR = inter-quartile range; LST = land surface temperature; N = number of observations; SES = socio-economic status.

* Median for continuous and proportion in percent for binary variables; IQR, minimum and maximum values only shown for continuous variables.

† According to Montresor, 1998 [21].

**Figure 3:**
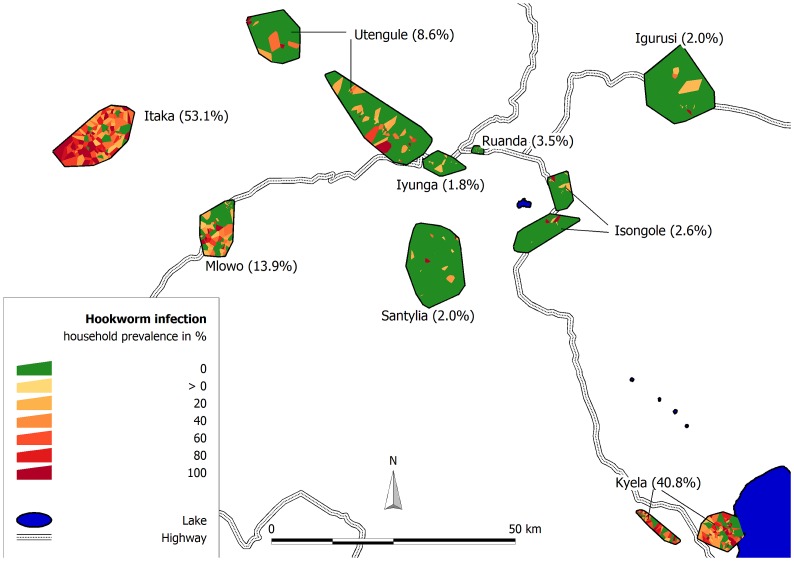
Hookworm prevalence in the EMINI study sites (Mbeya region, Tanzania, 2008/2009). Color shading of Voronoi polygons drawn around each household indicates household prevalence, labels indicate site name and site prevalence.

Site-specific hookworm prevalences ranged from less than 2% in Iyunga to more than 50% in Itaka (Figure 3). With 931 participants, Kyela was the biggest site and Iyunga with 444 participants the smallest. Due to exceptionally high hookworm prevalences, Itaka (53.1%) and Kyela (40.8%) were selected for the site-specific analyses.

### Univariable Logistic Regression and Multicollinearity Analysis

In univariable logistic regression analyses of the complete dataset which included all nine sites (Table 2) the estimates for all considered variables had p-values below 0.2, which was chosen as the cut-off for inclusion into further analyses. Population density, elevation and slope were inversely associated with hookworm infection, whereas the other ecological variables showed positive associations. SES, previous anthelmintic treatment, and latrine coverage were again inversely related to hookworm infection. Multicollinearity analysis revealed a VIF above 10 for the variables LST-night (VIF = 15.77), elevation (VIF = 75.18), and mean ambient temperature (VIF = 46.84). Elevation and mean annual ambient temperature were therefore excluded from subsequent analyses, and LST-night included since soil temperature seems more directly related to the development of hookworm larvae than ambient temperature and elevation. Removal of these two variables reduced the VIF for LST-night to 2.5.

**Table 2:** Univariable associations of considered variables with hookworm infection*.

Variable	OR	95% CI	p-value
**Ecological variables**			
Elevation [per 100 m]	0.87	0.85–0.89	<0.001
Mean annual EVI [per 0.1 units]	1.37	1.11–1.69	0.003
Mean annual LST-day [per 1°C]	1.19	1.14–1.24	<0.001
Mean annual LST-night [per 1°C]	1.24	1.21–1.27	<0.001
Mean annual ambient temperature [per 1°C]	1.37	1.32–1.41	<0.001
Mean annual rainfall [per 10 mm]	1.005	1.001–1.008	0.005
Slope [per 1°]	0.86	0.82–0.91	<0.001
Population density [per 1,000 persons/km^2^]	0.76	0.71–0.82	<0.001
**Adjustment variables**			
Age [per 10 years]	1.08	1.05–1.12	<0.001
Sex [0 = female/1 = male]	1.11	0.97–1.26	0.131
SES score [per 1 unit]	0.63	0.56–0.71	<0.001
Anthelmintic treatment in past year			
No	1.00		
Yes	0.44	0.30–0.64	<0.001
Missing	1.47	1.05–2.04	0.025
Latrine coverage [per 1%]	0.97	0.96–0.98	<0.001
Latrine type in household			
None	1		
Pit latrine simple	0.57	0.36–0.92	0.020
Ventilated improved pit latrine	0.15	0.05–0.44	0.001
Water flush toilet	0.12	0.03–0.48	0.003

CI = confidence interval; EVI = enhanced vegetation index; LST = land surface temperature; OR = odds ratio; SES = socio-economic status.

* From separate logistic regression models which only include one covariate, adjusted for household clustering using robust variance estimates.

### Model Selection

In the multivariable regression model including only household-level data (not shown), the p-values for mean annual rainfall, slope, and latrine type exceeded the 5% threshold and were excluded from the model. When including individual-level data into the model, only sex yielded a p-value above 0.05 and was excluded, whereas age and previous anthelmintic treatment remained significantly associated with hookworm infection. Compared to univariable regression results, the direction of the effect in the multivariable models changed for several variables: the ORs for EVI and LST-day switched from above to below unity; the negative univariable association of latrine coverage changed to positive in multivariable analysis.

In the multivariable model combining household-level and individual-level variables (Table 3, “All sites”) all included variables yielded significant p-values. No qualitative changes in the ORs compared to the separate models for household-level and individual-level variables (data not shown) were observed. Equally, the magnitude of effects in the combined model is comparable to those of the separate models, indicating that the effects of both sets of variables are independent of each other.

**Table 3:** Multivariable associations of selected ecological and adjustment variables with hookworm infection status*.

	All sites†			Kyela site			Itaka site			Moderated model‡		
	N = 6,372			N = 931			N = 846			N = 1,777		
Variables	OR	95% CI	p	OR	95% CI	p	OR	95% CI	p	OR	95% CI	p
Mean annual EVI [per 0.1 units]	0.11	0.06–0.18	<0.001	2.10	0.84–5.26	0.114	0.21	0.02–1.74	0.147	0.21	0.03–1.73	0.147
Mean annual LST-day [per 1°C]	0.81	0.75–0.88	<0.001	1.38	1.10–1.73	0.005	1.19	0.61–2.33	0.611	1.18	0.61–2.28	0.633
Mean annual LST-night [per 1°C]	1.54	1.44–1.64	<0.001	0.57	0.15–2.16	0.408	1.56	1.11–2.19	0.010	1.54	1.10–2.16	0.012
Population density [per 1,000 persons/km^2^]	0.68	0.63–0.73	<0.001	1.08	0.78–1.50	0.627	0.08	0.01–0.51	0.008	0.08	0.01–0.53	0.008
Age [per 10 years]	1.10	1.06–1.14	<0.001	1.12	1.04–1.20	0.002	1.16	1.06–1.28	0.001	1.14	1.07–1.20	0.000
SES score [per 1 unit]	0.82	0.73–0.93	0.002	0.68	0.50–0.93	0.015	0.79	0.58–1.09	0.158	0.79	0.57–1.08	0.143
Anthelmintic treatment [0 = n/1 = y]§	0.45	0.30–0.69	<0.001	0.73	0.36–1.46	0.376	0.42	0.18–0.97	0.042	0.52	0.29–0.94	0.030
Latrine coverage [per 1%]	1.02	1.01–1.04	0.002	0.99	0.96–1.02	0.484	0.94	0.89–0.99	0.022	0.94	0.89–0.99	0.022
Site [0 = Itaka/1 = Kyela]										3.26	0.04–243	0.592
EVI* Site										9.82	0.98–98.5	0.052
LST-day* Site										1.17	0.58–2.36	0.655
LST-night* Site										0.35	0.09–1.41	0.140
Population density* Site										12.79	1.97–83.2	0.008
SES score* Site										0.87	0.56–1.36	0.542
Latrine coverage* Site										1.05	0.99–1.12	0.086

CI = confidence interval; EVI = enhanced vegetation index; LST = land surface temperature; OR = odds ratio; SES = socio-economic status.

* Results of logistic regression adjusted for within-household clustering with robust variance estimates with each model containing only those variables for which data are shown in the Table.

† Performed on pooled dataset combining all nine sites.

‡ Moderated model for Kyela and Itaka sites with site-interaction terms for environmental variables.

§ Missing stratum not shown.

Running a model with the same variables on data from Kyela (Table 3, “Kyela site”) only yielded statistically significant ORs for LST-day (OR = 1.38; p = 0.005), SES score (OR = 0.68; p = 0.015), and age (OR = 1.12; p = 0.002). While the magnitude of ORs for SES score and age differed only marginally from the all-sites model, a qualitative difference was observed for LST-day where the association with hookworm infection switched from negative in the all sites model to positive in the Kyela site model. Site-specific analysis for Itaka (Table 3, “Itaka site”) resulted in significant ORs for population density (OR = 0.08; p = 0.008), LST-night (OR = 1.56; p = 0.010), latrine coverage (OR = 0.94; p = 0.022), age (OR = 1.16; p = 0.001), and prior anthelmintic treatment (OR = 0.42; p = 0.042), of which only latrine coverage differed qualitatively from the all-sites model.

To test the presence of site-specific effects, we introduced site-interaction terms for environmental variables. In the moderated model, which was estimated on a data set restricted to observations from Itaka and Kyela sites, only the interaction term for population density yielded a significant p-value (p = 0.008). The p-values for the interaction terms for EVI and latrine coverage slightly exceeded the 5% threshold (p = 0.052 and 0.086, respectively) and were therefore also considered as relevant. The main effects of the moderated model represent the effects in Itaka, i.e., site = 0, whereas the effects for Kyela (site = 1) can be calculated by multiplying the main effect with the respective effect of the interaction term.

**Figure 4:**
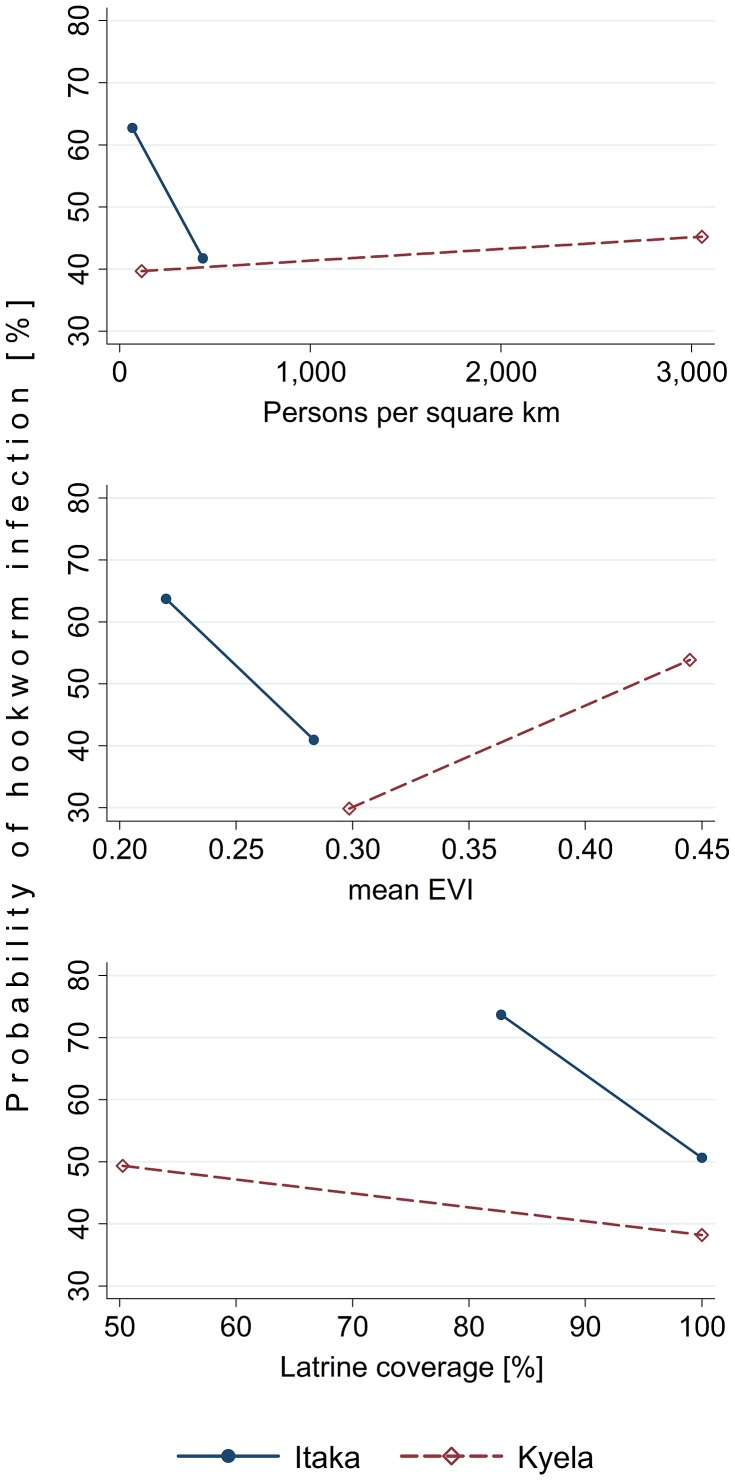
Linear predictions of hookworm infection probabilities for population density, EVI, and latrine coverage. According to the site-specific models for Kyela and Itaka, adjusted for all variables shown in the site-specific models in Table 3.

Keeping all variables at their average value, infection odds did not vary significantly between Kyela and Itaka (OR = 3.26; p = 0.592). However, the effect of population density, EVI and latrine coverage on infection odds was strongly dependent on the site. The data in Table 4 summarize the conditions in Kyela and Itaka, and the site-specific predictions of hookworm infection probability in Figure 4 demonstrate that a qualitative difference between the two sites was present for the association of population density and EVI with hookworm infection, whereas the association of latrine coverage differed only quantitatively between Itaka and Kyela sites.

**Table 4:** Characteristics of study participants and environmental conditions at their places of residence in Kyela and Itaka site.

	Kyela site					Itaka site				
Variable	N	Median or proportion (%)*	IQR	Min	Max	N	Median or proportion (%)*	IQR	Min	Max
Hookworm infection binary	931	40.8%				846	53.1%			
Hookworm infection intensity										
Median EPG (infected only)	380	144	276	12	5,012	449	144	228	12	4,772
Grouped†:										
No infection (0 EPG)	551	59.2%				397	46.9%			
Low (1–1,999 EPG)	375	40.3%				440	52.0%			
Moderate (2,000–3,999 EPG)	2	0.2%				8	1.0%			
Heavy (≥4,000 EPG)	3	0.3%				1	0.1%			
**Ecological variables**										
Mean annual EVI	931	0.373	0.060	0.299	0.445	846	0.249	0.016	0.220	0.283
Mean annual LST-day [°C]	931	32.1	3.2	27.8	35.0	846	34.4	0.6	33.1	35.4
Mean annual LST-night [°C]	931	21.1	0.3	20.6	21.4	846	15.0	1.0	12.9	16.2
Population density [persons/km^2^]	931	441	325	116	3053	846	214	167	68	437
**Adjustment variables**										
Age [years]	931	16.2	25.1	0.0	90.1	846	15.8	22.7	0.5	93.6
SES score	931	−0.59	1.16	−1.85	2.82	846	−0.18	0.84	−1.53	2.78
Anthelmintic treatment in past year	612	6.0%				840	5.5%			
Latrine coverage in surroundings [%]	931	89.5	15.5	50.3	100	846	100	0.0	82.8	100

IQR = inter-quartile range; EPG = eggs per gram of feces; EVI = enhanced vegetation index; LST = land surface temperature; N = number of observations; SES = socio-economic status.

* Median for continuous and proportion in percent for binary variables; IQR, minimum and maximum values are not given for binary variables.

† According to Montresor 1998 [21].

## Discussion

Our results demonstrate that hookworm infection in the study population is strongly associated with ecological factors. The univariable analyses further show that infection is favored when these factors entail more tropical conditions. This is in agreement with the literature, where similar associations of infection with elevation, temperature, rainfall, and vegetation (as an indicator of soil humidity and shade) are reported [22]–[24]. It also concurs with laboratory studies which show that hookworm larvae require warm and moist conditions in order to survive [25], [26], a fact that is also demonstrated by the absence of hookworm infection in more temperate climates world-wide [27] and very low prevalences in the high-altitude sites within our study area.

However, our data also show that some of these associations switch direction in multivariable analysis. The associations of EVI and LSTday with hookworm infection change from positive in univariable analysis to negative in the all-sites multivariable model, whereas the association of latrine coverage changes from negative to positive. These switches in direction are mainly due to the inclusion of LST-night, which appears to be the best predictor of hookworm infection among the environmental variables. When excluding LST-night from the all-sites multivariable model shown in Table 3, both EVI and LST-day maintain the significant positive association with infection (data not shown) that they have in univariable analysis (Table 2) and latrine coverage maintains its negative association, although this is no longer significant. For LST-day this makes sense in an area including high altitude sites with rather low temperatures. In this setting, the minimum temperature (for which LST-night is a better proxy than LST-day) is the main limiting factor for the survival of hookworm larvae. Therefore, in the complete model that includes both LST-night and LST-day, LST-night explains most of the variation that is due to unsuitably low minimum temperatures, whereas the role of LST-day in this model is limited to explain the variation that is due to unsuitably high maximum temperatures. In our study area, LST-day ranges from 22 to 39°C. Thus our finding corresponds with experimental study results suggesting that development of hookworm larvae reaches its peak between 20 and 30°C and ceases at around 40°C [25]. The switches in direction of the associations of EVI and latrine coverage with hookworm infection in multivariable analysis are harder to explain but are most likely based on similar effects. Contrary to the above described differences between univariable and multivariable models, population density, SES, age, and previous deworming show similar associations with hookworm infection in uni- and multivariable analyses. The negative associations of SES and deworming with infection are highly plausible and have also been reported in other studies [1], [28]. This also applies to the positive association of age with infection [29], [30].

Regarding the relationship of population density with infection, the interpretation is more complicated. While higher population densities increase the chance of hookworm larvae to find a host and could thus favor transmission, in our study area, they are also an indicator of more urban and thus more developed conditions, which would reduce transmission. Thus, the negative association with hookworm infection found in this study is also plausible, and accordingly both negative and positive associations have been reported in the literature [10], [24].

Another interesting phenomenon are the differences in association of several factors, when comparing the two site specific multivariable models for Kyela and Itaka (columns “Kyela site” and “Itaka site” in Table 3) with each other and when comparing each of them with the all-sites model (“All sites” in Table 3). When comparing Tables 1 and 4, it is obvious that ecological variables in the all-sites model cover a much wider range of conditions than in each site-specific model, which is a plausible reason for the differences of the site-specific models *versus* the all-sites model.

Figure 4 demonstrates that the above reasoning may also apply to the contradictory results when comparing the two site-specific models with each other: population density and latrine coverage show far more variation in Kyela than in Itaka, and the EVI ranges for both sites do not show any overlap, with much lower vegetation cover in Itaka. Thus, the different conditions in the two sites are a likely explanation for the different associations of these factors in the two sites. However, it is also possible that these differences in association are a consequence of one or more unobserved factors that our analysis is unable to account for.

Strengths of this study include the large number of participants of all age groups and the detailed information that we have for each individual, including the place of residence. This allows for a detailed assessment of individual exposure to environmental factors. In contrast, most other studies into the spatial epidemiology of hookworm and other soil-transmitted helminth infections are school-based [9]–[11], [24], [31]–[33]. Thus, they do not examine hookworm infection in adults and rely on the geographical position of the school to quantify participant's exposure to environmental factors [34].

However, our study also has some limitations. The use of only one stool specimen for the determination of hookworm infection status is known to lack sensitivity due to the intra-specimen and day-to-day variation in hookworm egg output [35], [36]. Although we prepared two Kato-Katz thick smears from each stool specimen to increase sensitivity, it is likely that we missed some of the lighter infections. Unfortunately the Kato-Katz examination of stool is unable to differentiate between *N. americanus and A. duodenale*. However, previous studies indicate that *N. americanus* is the predominant species in East Africa [32], and stool-PCR data from our own ongoing WHIS study, where we only find *N. americanus* infections, seem to indicate that this is also the case for our study area. Thus it is likely that most or all of the hookworm infections in our study population were caused by *N. americanus*, although we cannot completely exclude that *A. duodenale* is also present.

Unfortunately, we are also lacking information about soil composition in the study area which has been shown to strongly influence hookworm infection [24], [31]. Motility of the hookworm larvae is crucial to avoid adverse environmental conditions and is thus important for their survival. The porosity of sandy soils facilitates larval movement deeper into the soil to escape desiccation and upwards movement to avoid rising water levels after heavy rainfall. Soils with high clay content are less porous and thus inhibit larval motility [23], [37]–[39].

Furthermore, apart from previous worm treatment which was assessed by interview, our study does not account for behavioral factors which also can strongly influence hookworm transmission and prevalence. However, although soil composition and behavior are both important determinants of hookworm infection which would likely have improved our models if included, data on these factors are rarely available in tropical developing countries where hookworm is most prevalent. Thus, their potential to predict infection in order to plan helminth control is limited, especially in those regions where control is urgently needed.

This study and many others have shown that remotely sensed data such as vegetation indices, LST, and elevation are strongly associated with hookworm prevalence [2], [8]. However, our study also shows that these associations are scale-dependent and that predictions using these data should be handled with care. On a large scale, they can provide powerful tools to identify regions that warrant control and intervention programs, their big advantage being public availability and global coverage.

Nevertheless, when making predictions of hookworm infection on a smaller scale, regional characteristics, such as seasonal flooding, dry spells, etc., have to be taken into account. As our study has shown, even within a relatively small geographic area the effects of environmental conditions can differ to a large extent. Thus, large-scale findings cannot necessarily be used for prediction on smaller scales and *vice versa*.

## Supporting Information

Checklist S1 **STROBE checklist.**
(DOC)
